# Single Nucleotide Polymorphisms in the Promoter Region of *MyoG* Gene Affecting Growth Traits and Transcription Factor Binding Sites in Guizhou White Goat (*Capra hircus*)

**DOI:** 10.3390/genes17010014

**Published:** 2025-12-25

**Authors:** Xingchao Song, Huaixin Long, Jinzhu Meng, Yuanyuan Zhao, Zhenyang Wu, Qingming An

**Affiliations:** Guizhou Provincial Key Laboratory for Biodiversity Conservation and Utilization in the Fanjing Mountain Region, College of Agriculture and Forestry Engineering, Tongren University, Tongren 554300, China; songxingchao_888@126.com (X.S.); 19110826607@163.com (H.L.); mjz122821@126.com (J.M.); yjy13251252247@163.com (Y.Z.); wuzhenyang0724@163.com (Z.W.)

**Keywords:** Guizhou White goat, *MyoG* gene, promoter region, SNPs, transcription factor

## Abstract

**Objective**: Growth traits are important economic characteristics in livestock. Genetic polymorphism has great influences on the improvement of goat growth traits. As an important member of the myogenic regulatory factor (MRFs) family, *MyoG* gene polymorphisms can alter the growth characteristics in goats. In this study, we aimed to investigate the regulation mechanism of the *MyoG* gene promoter region from the perspective of single nucleotide polymorphisms (SNPs) and transcription factors. **Methods**: Genomic DNA sequencing was carried out to detect SNPs in the −1000 bp upstream to 300 bp downstream of the *MyoG* gene promoter region in 224 Guizhou White goats (*Capra hircus*), and the genetic parameters of novel SNPs were calculated. The association between SNPs and growth traits, comprising body weight, body length, body height, chest circumference and cannon circumference, were analyzed using one-way ANOVA by IBM SPSS 23.0 software according to the general linear model. Transcription factor binding sites in the promoter region of the *MyoG* gene before and after mutation were predicted using bioinformatics software programs. **Results**: Four SNPs, including g.–709C>T, g.–461G>T, g.–377G>T and g.–249G>A, were identified in the 1 246 bp promoter region of the *MyoG* gene in Guizhou White goats. Based on χ^2^ test, the g.–709C>T and g.–461G>T loci were consistent with Hardy–Weinberg equilibrium, while two other SNPs were deviated from Hardy–Weinberg equilibrium in Guizhou White goats. Association analysis revealed that the body weight of those with the CT genotype at the g.–709C>T locus was greater than of those with the CC and TT genotypes in Guizhou White goats (*p* < 0.05). At the g.–461G>T locus, the body weight of individuals with the GG genotype was significantly higher than that of those with GT genotype (*p* < 0.01). The body length of individuals with the GG genotype formed by the g.–249G>A locus was significantly higher than that of those with the GA genotype (*p* < 0.01). Online software programs found that four SNPs within the promoter region of the *MyoG* gene changed some transcription factor binding sites. **Conclusions**: Mutations of the *MyoG* gene promoter region may have a significant regulatory effect on the growth traits of Guizhou White goats. The small sample size may be one of the limitations for this study; nevertheless, these findings could provide a theoretical basis for further exploring the relationship between the four SNPs studied and the growth traits in Guizhou White goats, as well as the promoter function of the *MyoG* gene.

## 1. Introduction

Myogenic regulatory factors (MRFs) are known as vital members of the myogenesis regulatory family, which is involved in the proliferation of muscle precursor cells and in the formation of muscle fiber, as well as in postnatal muscular functions [1,2]. Previous studies have shown that the *myogenin* (*MyoG*) gene is the only member in the family of MRFs that can be expressed in animal skeletal muscle cell lines. It can activate muscle gene transcription, improve cell differentiation and is a key factor in regulating skeletal muscle growth and development. It plays a central regulatory role in the process of muscle cell generation [3]. At the same time, the *MyoG* gene interacts with other transcription regulatory factors in the MRFs family, such as *MyoD*, *Myf5* and *MRF4*, to jointly regulate animal meat quality traits and meat yield [4]. Thus, genetic variation in the *MyoG* gene may affect the growth, carcass and meat traits in livestock. In previous studies, some MRFs were confirmed to be related to meat quality traits in swine [5,6], beef cattle [7] and sheep [8,9]. In sheep, variants in *MyoD1* were reported as being associated with body traits such as thoracic girth and loin width [10]. Additionally, positive correlations between *MyoG* expression and body weight in Hu sheep were explored [11]. At present, candidate gene analysis has been achieved using relevant molecular methods [12], and the candidate gene method has been proven to be effective in identifying gene polymorphisms. If the genetic variation in quantitative traits in organisms is caused by causal mutations in candidate genes, it can be determined from this perspective that the analysis of gene physiology, biochemical function and metabolic pathways is helpful in selecting potential candidate genes for evaluation.

Guizhou White goats, with advantages such as strong adaptability, stable genetic performance, fresh flesh and having a delicious taste, make for an important germplasm resource of animal husbandry in the Guizhou province, China [13]. While being smaller in size, growing more slowly, and having lower meat production rates are contributing factors for the low economic efficiency of Guizhou White goats [14,15]. The growth characteristics of goats have high economic value, and increasing lamb yield in practical production largely depends on identifying and exploring how genetic variations in key genes that control growth rate regulate gene expression at the molecular level.

In recent years, the Genome-wide association study (GWAS) has gradually been applied to conduct research on goat body weight, body size and meat quality traits, identifying a large number of important genes and their associated SNPs for quantitative and qualitative traits, including Zhongwei goat [16], Karachai goat [17], Youzhou Dark goat [18] and Sub-Saharan Africa indigenous goat [19], enriching the scope of goat genomics research. However, there are relatively few SNPs screened in the promoter region, especially key factors related to gene transcription regulation mechanisms. Promoters are important regions for gene regulation, as they contain many sites and functional domains that bind to transcription regulatory factors. Therefore, the polymorphic sites in these regions are likely to affect the mRNA expression level of the gene to some extent, further affecting the protein function and causing changes in animal phenotypic traits.

So far, research on the relationship between the promoter region of the *MyoG* gene and growth traits is limited. The main objective of this study is to further investigate the association between novel SNPs within the promoter region of the *MyoG* gene and body size in goats. The *MyoG* gene in goats is located on chromosome 16, and there are several quantitative trait loci (QTLs) related to carcass weight [20]. Up to now, there have been no reports on the association between genetic variations in the promoter region of the *MyoG* gene in goats and growth traits. Thus, this study aims to explore the SNPs in the promoter region of the *MyoG* gene in Guizhou White goats and their effects on body measurement traits and transcription factor binding sites.

## 2. Materials and Methods

### 2.1. Statement of Ethics

The collection of goats samples involved in this study has been approved by the Ethics Committee of Tongren University, China (Permit number for conducting animal experiments: TRXY 2023-088; Approval date: 8 March 2023), and everything possible was done to minimize the pain of the goat as much as possible.

### 2.2. Animal Sources, Data Collection and DNA Extraction

Male Guizhou White (GZW) goats (*n* = 224 of about three years of age) were selected at random from Huazhen Animal Husbandry Co., Ltd. in Tongren City, Guizhou Province, China. According to farm records, all of the goats were GZW goats that were genetically distinct from each other and were subjected to the same environmental conditions and nutrition. According to the same standards, peripheral blood samples (5 mL) were collected from each goat through the jugular vein using a vacuum tube and frozen at −20 °C for DNA extraction. The growth traits of all selected goat individuals have been recorded by the breeding farm. Body size indicators include the following: body weight (BW), body length (BL), body height (BH), chest circumference (ChC) and cannon circumference (CaC). The blood genomic DNA of Guizhou White goats was extracted with the blood genomic DNA extraction kit (TransGen Biotech, Beijing, China), and its concentration and purity were detected by ultraviolet spectrophotometer and 0.7% agarose gel electrophoresis diluted to 80 ng/μL and stored at −20 °C.

### 2.3. Primer Design and PCR Amplification

Based on the flanking regions of the *MyoG* gene in *C. h.* (Accession number FJ607135) and *Bos taurus* (Accession number HM452338) as reference sequences, a pair of specific primers (Forward: 5′-CTTCCCTCCTCACCCACATT-3′, Reverse: 5′-TCCACAGACACCGACTTCCT-3′) were designed using Oligo 7.0 and Primer Premier 5.0 software to amplify the promoter region of the *MyoG* gene in Guizhou White goats. The primers were synthesized by Sangon Biotech Co., Ltd. (Shanghai, China). PCR amplification was performed in 40 μL reaction mixture, which was made up of 20 μL 2 × SanTaq Fast Mix (Sangon Biotech Co., Ltd., Shanghai, China), 1.0 μL of 80 ng/μL template DNA, 1.0 μL of 10 pmol/μL each primer and 17 μL sterilized ultrapure water. The PCR thermal cycle was programmed as follows: pre-denaturation at 94 °C for 5 min, denaturation at 94 °C for 30 s, annealing at 58.8 °C for 30 s, extension at 72 °C for 75 s and 40 cycles and a final extension at 72 °C for 10 min, stored at 4 °C. PCR products were detected by 1.2% agarose gel electrophoresis and then sent to Sangon Biotech Co., Ltd. (Shanghai, China) for sequencing. SNPs in the MyoG gene were detected by bidirectional direct sequencing.

### 2.4. SNPs Screening and Genotype Determination

The obtained *MyoG* gene sequences of 224 Guizhou White goats were aligned by the ClusterW Multiple alignment program in BioEdit 7.0 software and detected SNPs sites. The genotype of SNPs loci was determined using the sequencing peak mapping software Chromos 5.0, with a single peak representing a homozygous genotype and a nested peak representing a heterozygous genotype.

### 2.5. Genotype, Allele Frequency and Association Analysis Between SNPs Loci and Growth Traits

The genotype, allele frequency and Hardy–Weinberg equilibrium (HWE) of SNPs loci were analyzed based on the online software SHEsis (http://analysis.bio-x.cn, accessed on 8 June 2024). Polymorphism information content (*PIC*) was calculated through online software (http://www.msrcall.com/Gdicall.aspx, accessed on 18 June 2024). The mean and standard error of the different growth traits of 224 individual Guizhou White goats with different SNPs loci of the *MyoG* gene were statistically analyzed by means of IBM SPSS 23.0 software. At the same time, according to the general linear model, which is used to make a significant test on the effects of various genotypes on growth traits, one-way ANOVA was used to conduct a homogeneity of variance test and a multiple comparison test and to further analyze the correlation between SNPs locus and growth traits of Guizhou White goats. A general linear model is y_ij_ = μ + α_i_ + ε_ij_, where y_ij_ is the individual growth trait (body weight, etc.) record, μ is population mean for each growth trait, α_i_ is the genotype effect and ε_ij_ is the random error. The data is displayed as mean ± standard error (SE), with *p* < 0.05 indicating statistically significant differences and *p* < 0.01 indicating extremely significant differences.

### 2.6. Identification of Transcription Factor Binding Sites Before and After SNPs Mutation

The possible transcription factor binding sites in the promoter region of the *MyoG* gene in Guizhou White goats were predicted using AliBaba 2.1 software (https://gene-regulation.com/pub/programs/alibaba2/index.html, accessed on 6 July 2024). Predicting changes in transcription factors before and after SNPs mutation in the promoter region of the *MyoG* gene by means of a transcription factor binding database profile (JASPAR^2024^: https://ngdc.cncb.ac.cn/databasecommons/database/id/9171, accessed on 10 August 2024).

## 3. Results

### 3.1. Detection of MyoG Gene PCR Products

**Figure 1:**
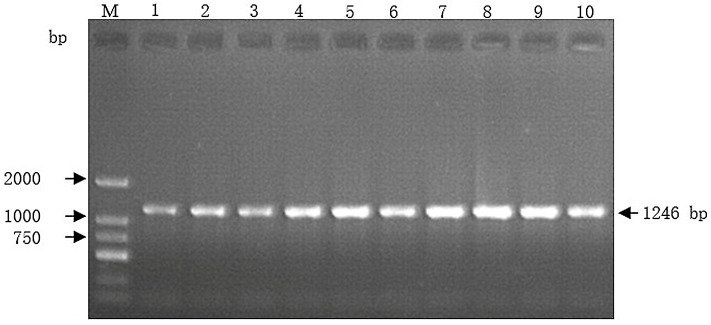
Agarose electrophoretic map of the *MyoG* gene specific fragment in Guizhou White goats. Lane M represents DL 2000 Marker. Lane 1 to 10 denotes target segment. The arrow indicates the 1246 bp of the *MyoG* gene fragment.

The designed primer was used to amplify the *MyoG* gene of Guizhou White goats, and the PCR products were separated by 1.2% agarose gel electrophoresis. As shown in Figure 1, the size of the amplified fragment is consistent with the target fragment. The amplified band is clear and dense, which could be used for subsequent direct sequencing analysis.

### 3.2. SNP Identification in the Promoter Region of the MyoG Gene

**Figure 2:**
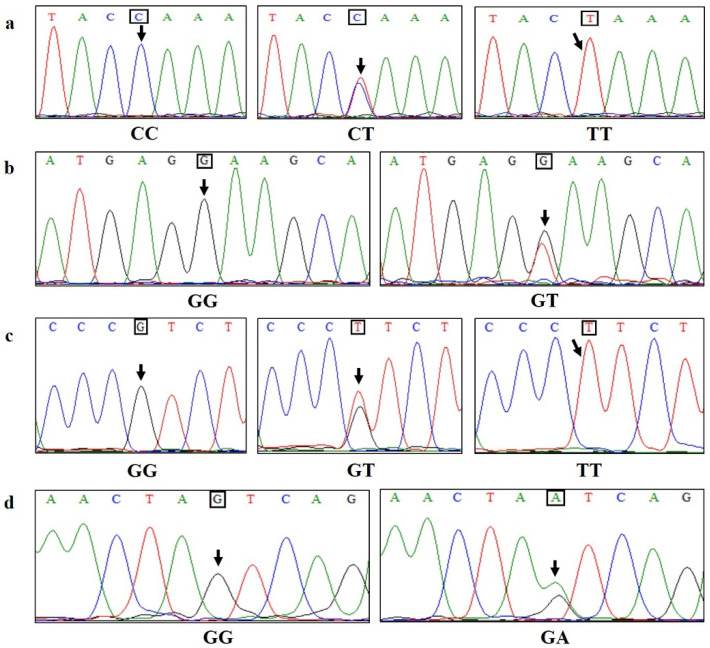
The sequencing chromatogram of four SNPs for the *MyoG* gene in Guizhou White goats; SNPs are indicated by arrows. (**a**–**d**) represent sequencing results of each genotype at g.–709C>T, g.–461G>T, g.–377G>T and g.–249G>A, respectively.

Based on the sequence structure of the bovine *MyoG* gene in the GenBank database, a total of 1246 bp nucleotide sequences of the *MyoG* gene in Guizhou White goats were obtained, including 1004 bp 5′UTR and 242 bp exon 1. The Clustal W Multiple alignment program in BioEdit 7.0 software was used to compare the sequencing results of 224 Guizhou White goats, screen for SNPs and determine the genotype of each locus based on the sequencing peak maps. The naming principle of SNPs is that the A base in the start codon ATG is marked as “+1”. At the same time, the obtained 1246 bp sequence was aligned with the goat genome reference sequence in the NCBI database, using BLAST (version 2.17.0) to determine the specific location of the identified SNPs in the goat chromosome. The *MyoG* gene is located on chromosome 16 of Guizhou White goats. Four SNPs were screened in the promoter region of the *MyoG* gene in Guizhou White goats, including g.–709C>T, g.–461G>T, g.–377G>T and g.–249G>A, which are located at 193,987 bp, 193,739 bp, 193,655 bp and 193,528 bp of the goat reference genome sequence NC_030823.1, and the variant IDs are rs666139870, rs636117637, rs652431992 and rs639389894, respectively. Two genotypes were formed at the g.–461G>T and g.–249G>A loci in Guizhou White goats, while three genotypes were found at the g.–709C>T and g.–377G>T loci. The sequencing results of the four SNPs are shown in Figure 2 and Supplementary Materials, Figure S1.

### 3.3. Polymorphic Parameter, Genotype and Allele Frequencies for Different SNPs

The genotype and allele frequencies of *MyoG* gene SNPs in Guizhou White goats are shown in Table 1. Three genotypes were found at the g.–709C>T locus, namely, CC, CG and GG; GG, GT and TT were identified at the g.–377G>T locus. Two genotypes were found at the g.–461G>T locus, namely, GG and GT; GG and GA were screened at the g.–249G>A locus. The χ^2^-test showed that Guizhou White goats conform to the Hardy–Weinberg equilibrium at the g.–709C>T and g.–461G>T loci (*p* > 0.05), while the g.–377G>T and g.–249G>A loci significantly departed from the Hardy–Weinberg equilibrium (*p* < 0.01). At the g.–709C>T, g.–461G>T, g.–377G>T and g.–249G>A loci in the promoter region of the *MyoG* gene, the frequencies of the C, G, G and G alleles are higher than those of alleles T, T, T and A. As shown in Table 1, the population genetic parameters of four SNPs of the promoter region of the *MyoG* gene in Guizhou White goats were calculated. The *PIC*, *Ne*, *Ho* and *He* of the g.–709C>T locus were 0.36, 1.92, 0.52 and 0.48 and of the g.–377G>T locus were 0.29, 1.53, 0.65 and 0.35, respectively, indicating that Guizhou White goats have moderately polymorphisms (0.25 < *PIC* < 0.5) at the g.–709C>T and g.–377G>T loci of the *MyoG* gene promoter region.

### 3.4. Association of MyoG Gene Promoter Region Polymorphism and Growth Traits in Guizhou White Goats

In order to investigate the association between the above mentioned SNPs and the growth traits of Guizhou White goats, a general linear model was used for association analysis by means of IBM SPSS 23.0 software. As shown in Table 2, the individuals with the CT genotype formed by the g.–709C>T locus in the promoter region of the *MyoG* gene in Guizhou White goats had significantly higher body weight than those with the CC and TT genotypes (*p* < 0.05), and the chest circumference of individuals with CT genotype was significantly higher than that of individuals with the CC and TT genotypes (*p* < 0.05). The body length, body height and circumference of individuals with the CT genotype tended to be higher than that of those with the CC and TT genotypes, but these differences are not significant (*p* > 0.05). The body weight of individuals with the GG genotype caused by the g.–461G>T locus was extremely significantly higher than those with the GT genotype (*p* < 0.01), and no significant differences were observed in body length, height, chest circumference or cannon circumference among individuals with different genotypes. (*p* > 0.05). No significant differences were observed in body weight, body length, body height, chest circumference or cannon circumference among genotypes at the g.−377G>T locus (*p* > 0.05). The body length of individuals with the GG genotype caused by the g.–249G>A locus was significantly higher than those with the GA genotype (*p* < 0.01), and no significant differences were observed in body weight, body height, chest circumference and cannon circumference among individuals with different genotypes (*p* > 0.05).

### 3.5. SNPs in the Promoter Region of the MyoG Gene Cause Alterations in Transcription Factors

The alterations of transcription factors in the *MyoG* gene promoter region, calculated by the online software JASPAR^2024^, are shown in Table 3. The g.–709C>T locus may result in the vanishing of both the C/EBPalp and serum response factor (SRF) transcription factor binding sites and in the increase of a new TATA binding protein (TBP) site. The g.–461G>T locus may result in the vanishing of the PU.1 transcription factor binding sites and in the increase of activator protein 1 (AP-1). The g.–377G>T locus may lead to the vanishing of the specific protein 1 (SP1) transcription factor binding sites and to the increase of octamer binding transcription factor 1 (OCT-1) binding sites. The g.–249G>A mutation site may result in the vanishing of the ELA1 transcription factor binding site and in the increase of ALX1 binding sites. Thus, these SNPs, especially at newly identified loci, may form new transcription factor binding sites while disrupting primitive existing binding sites and disturbing transcriptional regulation and expression of the *MyoG* gene.

## 4. Discussion

### 4.1. Correlation Between MyoG Gene SNPs and Growth Traits of Caprinae

The Guizhou White goat is an excellent local breed in the Guizhou province, with advantages such as strong adaptability, tolerance to rough feeding, tender meat and delicious meat taste. However, this breed has disadvantages such as small size, low slaughter rate, low net meat rate and slow growth rate, which limit the large-scale breeding and industrial development of local goat breeds. Screening candidate genes and related molecular markers for goat growth traits at the molecular level and conducting marker-assisted selection breeding will be one of the effective means to improve goat meat production capacity and meat quality. It is also a good way to improve local goat breeds with Guizhou characteristics or cultivate new meat breeds [21]. There are few SNPs in the *MyoG* gene of goats, all of which are significantly correlated with meat quality and growth traits, indicating that the *MyoG* gene is a key candidate gene for mutton development [22]. Screening of genetic variant molecular markers of the *MyoG* gene associated with growth traits is of significant theoretical importance and has implications for the development of molecular breeding or meat production traits. At present, research on *MyoG* gene SNPs is mainly focused on pigs [23,24,25], cattle [26,27] and sheep [28,29], mainly involving the association between SNPs and meat quality and carcass traits. Previous studies have analyzed sheep *MyoG*, including three exons and two introns, and identified some polymorphisms. Han et al. [30] reported that there was one single nucleotide missense mutation of A109C at exon 1 of a Tibetan sheep *MyoG* gene. Statistical analysis indicated that in the Henan Oula Tibetan sheep population, individuals with the CC genotype have higher mean values for body weight, body height and body length than those with the AA genotype. Li et al. [9] analyzed the polymorphism in intron 2 of *MyoG* gene in Chinese sheep local breeds using the PCR-RFLP method. The dominant genotype was AB, and the *Eco* 72 I locus could serve as a molecular marker affecting sheep growth traits. The same SNPs of the *MyoG* gene intron 2 have also been reported in Boer goat populations, which indicates that the *MyoG* gene has a certain influence on the growth traits of goats [31]. Thus, investigating the SNPs in the promoter region of the *MyoG* gene and their associations with growth traits in Guizhou White goats represents a significant approach for discovering new molecular genetic markers used in goat breeding practices.

Previous studies have found that *PIC* value is an important parameter for marker site polymorphism in linkage analysis [32,33]. In this study, four SNPs in the promoter region of the *MyoG* gene were identified, and the relationship between SNPs and growth traits in Guizhou White goats was studied. These SNPs appear to be new and have never appeared in previous databases. In addition, their impact on the *MyoG* gene expression or their association with goat growth traits have not been explored. For the g.–709C>T and g.–377G>T loci, the *PIC* value has been obtained and were identified as moderate polymorphic in the Guizhou White goat population (0.25 < *PIC* < 0.5). This indicates that breeders can utilize significant genetic diversity throughout the entire evolutionary history of animals to select superior varieties. For the g.–461G>T and g.–249G>A loci, the *PIC* value exhibits low level polymorphism. Simultaneously, the goat population in this study was in Hardy–Weinberg equilibrium at the g.–709C>T and g.–461G>T loci. This equilibrium may be due to the stable genetic status of the Guizhou White goat population after long-term breeding, which suggests that selective breeding measures may be taken in the future to improve production traits such as growth rate, meat quality and carcass traits. Nevertheless, the g.–377G>T and g.–249G>A loci exhibited a significant deviation from the Hardy–Weinberg equilibrium, which may be due to an insufficient sample size that does not adequately represent the phenotypic distribution of these loci within these broader populations. Therefore, it is imperative that the Guizhou White goat sample size be expanded in future studies to validate this discovery.

The CT genotypes of the g.–709C>T locus appear to have a relatively higher impact on body weight and chest circumference in Guizhou White goats (*p* < 0.01). The genetic variation at this locus is directly associated with the weight and chest circumference of Guizhou White goats, which may involve dominant effects or heterozygous advantages. Similarly, in Nile tilapia, heterozygosity at certain SNP loci of the *MYF5* gene is significantly associated with growth traits [34]. Therefore, heterozygous state may enhance growth potential through gene interactions. The significant effect of heterozygous genotypes suggests that these SNPs may affect growth traits through dominant or heterozygous dominance, providing a key target for genetic improvement. This result suggests that screening heterozygous genotypes in molecular marker-assisted breeding of goats has important theoretical significance and practical value, which is helpful for breeding excellent meat breeds of Guizhou White goats with rapid growth. The GG genotypes of the g.–249G>A locus appear to have a relatively higher impact on body length in Guizhou White goats (*p* < 0.01). The GG genotypes of the g.–461G>T locus appear to have a relatively higher impact on body weight in Guizhou White goats (*p* < 0.01).

Nevertheless, it must be clarified that body weight, chest circumference and body length are quantitative traits determined by many genes, including *MYOD1*, *MSTN*, *IGFs* [35,36,37] and so on. Therefore, the impact of single genes and mutations may be minimal. In addition, the application of *MyoG* gene polymorphism in Guizhou White goat breeding may be hindered by bias in genotype and allele distribution, even lacking some genotypes in goat populations (such as the TT genotype of the g.–461G/T locus). Therefore, their value in breeding and selection must still be validated on sufficient goat populations, preferably on different goat breeds. In addition, the next step of this study in the future should be to expand the sample size of goats and further analyze the association between SNPs haplotypes in the *MyoG* gene promoter region and goat growth traits.

The SNPs identified in the untranslated region and exons of the *MyoG* gene may have a significant impact on the expression of this gene. In Jinghai yellow chickens, individuals with the BB genotype of the *MyoG* gene, which was formed by g.36T>C mutation in exon 3, had higher bodyweight at different weeks [38]. In zebrafish embryos, the expression of the *MyoG* gene is regulated by lots of regulatory elements, especially multiple transcription factors in the promoter region [39]. Current research has only analyzed the correlation between SNPs and growth traits of Guizhou White goats. The correlation analysis between SNPs and the expression level of this gene will be the focus of our subsequent research. However, as far as we know, this study is the first to investigate the association between SNPs in the promoter region of the *MyoG* gene and growth traits in goats.

In the screening of SNPs in the exons and introns of the *MyoG* gene in goats, individuals with the AA genotype formed by the g.1823C>T mutation in intron 2 had significantly lower birth weight than individuals with the AB and BB genotypes. Selecting individuals with the B allele is expected to improve growth traits related to goat body mass [31]. In terms of other members of the myogenic regulatory factor family, myogenic differentiation factor 1 is also a gene locus significantly associated with growth traits. The Copy Number Variation loci containing the *MyoD1* gene exon in Shanbei white cashmere goats is significantly correlated with body weight, body height, hip height, chest circumference and hip circumference (*p* < 0.05), indicating that this locus is a marker-assisted selection breeding locus for goats [35]. In addition, insulin-like growth factor 1 (*IGF-1*) is an important member of the animal growth axis, mainly regulating fetal growth and development, and is a key factor for linear growth in animals after birth. Several QTLs related to growth and body shape traits were identified on goat chromosome 5, including the *IGF-1* gene [40]. Many researchers have directly sequenced different goat breeds and found a large number of SNPs in the 5′untranslated region of the goat *IGF-1* gene, some of which are significantly correlated with growth traits such as birth weight, chest width and chest depth [41,42]. However, whether the SNPs of the *MyoG* gene discovered in this study jointly affect the growth traits of Guizhou White goats with the above mentioned mutation sites still needs further verification through subsequent gene interaction and related analyses.

In future research, we will expand to genome-wide association analysis (GWAS) to systematically identify more SNPs loci related to the growth traits of Guizhou White goats. In addition, it is necessary to combine functional validation experiments, such as gene editing or expression analysis, to clarify the biological mechanisms of candidate genes. Strengthening the integration of genetic resource protection and molecular breeding techniques will help achieve precise breeding for Guizhou White goats.

### 4.2. Effects of Transcription Factor Binding Sites Alteration in the Promoter Region on Transcriptional Regulation of MyoG Gene

The promoter region is a specific DNA sequence located upstream of a gene, which is a key and important region for RNA polymerase and transcription factors to bind and initiate gene transcription. SNPs in the promoter region may regulate gene expression by altering the binding mode of some transcription factors, which is one of the important mechanisms for understanding individual genetic differences and susceptibility to complex diseases [43,44]. SNPs in the promoter region may indeed alter transcription factor binding sites by directly disrupting or creating transcription factor binding sites and indirectly affecting binding affinity or conformation, thereby affecting gene expression [45,46].

In this study, the transcription factor prediction of the MyoG gene promoter region showed that the mutation of g.–709C>T resulted in the disappearance of the initial C/EBPalp and SRF transcription factor binding sites and in the formation of a new binding site for the TBP transcription factor. TATA binding protein (TBP) is essential for RNA polymerase III transcription. Previous studies have found that TBP plays a central role in transcriptional initiation, which was first discovered to recognize and bind to the TATA box in the gene promoter region. In addition, TBP is a key factor necessary for initiating the three major eukaryotic RNA polymerases [47,48]. Regarding the newly added TBP transcription factor binding sites, this study only used bioinformatics software for predictive analysis. Whether there is a positive over-effect still needs to be verified in the future. Similarly, the sample size should also be increased for analysis in the future.

The mutation of g.–461G>T locus resulted in the vanishing of the initial PU.1 transcription factor binding site and in the increase of a new binding site for the AP-1 transcription factor. Activation protein-1 (AP-1) is an inducible transcription factor which is composed of multiple protein complexes, including members of the fos and jun gene families. Many cells and viral genes contain AP-1 binding sites in their promoters; therefore, AP-1 has been investigated to determine whether it plays a role in regulating these genes and inducing transcription [49]. Further in-depth research is needed to determine whether the increased AP-1 transcription factor caused by the g.461G>T mutation in the promoter region of the *MyoG* gene in Guizhou White goats will affect the transcription and functional expression of this gene.

The mutation of g.–377G>T locus, led to the vanishing of the initial SP1 transcription factor binding site; meanwhile, a new binding site for the OCT-1 transcription factor was raised. OCT-1 was first found as a member of the POU transcription factor family and is widely expressed in most cells and tissues. OCT-1 plays an extremely important role in development and differentiation processes [50]. The specific role of the increased OCT-1 transcription factor in the promoter region of the *MyoG* gene in Guizhou White goats also requires further verification and exploration. The mutation of g.–249G>A locus resulted in the disappearance of the initial ELA1 transcription factor binding site and a new binding site for the ALX1 transcription factor was formed. Alx1 is a transcription factor containing homologous domains and is a highly conserved regulatory factor for bone formation in echinoderms. In sea urchins, Alx1 plays a central role in the differentiation of embryonic primary mesenchymal cells (PMCs) and actively regulates the transcription of most biomineralization genes expressed in these cells [51]. The function of the Alx1 transcription factor in the development of muscle and bone tissue in vertebrates, especially goats, also needs to be further explored.

These four SNPs in the promoter region of the *MyoG* gene have altered the initial transcription factor interaction, which may, therefore, be leading to alterations in promoter activity and gene function. Alterations of the transcription factor are likely to decrease or increase the expression level of the *MyoG* gene, which indirectly affects the growth traits of Guizhou White goats. Thus, it is speculated that the alterations of transcription factors may affect the meat production capacity of Guizhou White goats, but the specific mechanism still needs to be explored further in-depth.

## 5. Conclusions

In the current study, the *MyoG* gene promoter region sequences of 224 Guizhou White goats were explored. Four SNPs were screened in the region –1000 bp upstream to 300 bp downstream of the goat *MyoG* gene transcription start site. The mutation within the promoter region is being reported for the first time in goats. Association analysis suggests that g.–709C>T and g.–461G>T loci in the promoter region are molecular marker sites that affect the weight index of Guizhou White goats. The g.–249G>A site is a molecular marker site that affects the body length index of Guizhou White goats. These SNPs might lead to alterations in their initial transcription factor binding sites, thus potentially changing gene expression. However, the relationship between the SNPs identified in this research and the expression level of *MyoG* gene in goats should be further studied in the future. Similarly, we will use a dual luciferase reporter gene vector to detect the promoter activity and determine the promoter active region of Guizhou White goats and verify the transcription factors of the mutation sites through EMSA or Chip methods. In short, these findings provide a theoretical basis for further exploring the regulation mechanisms of *MyoG* expression and its relationship with goat breeding.

## Figures and Tables

**Table 1:** Population genetic analysis of four SNPs of the *MyoG* gene promoter region in Guizhou White goats.

Mutation Site	Genotype Frequency ^1^			Allele Frequency		χ^2^	*p*-Value	*PIC*	*Ne*	*Ho*	*He*
g.–709C>T	CC	CT	TT	C	T	1.23	0.54	0.36	1.92	0.52	0.48
	0.38 (85)	0.45 (100)	0.17 (39)	0.60	0.40						
g.–461G>T	GG	GT	TT	G	T	1.24	0.27	0.12	1.15	0.87	0.13
	0.86 (193)	0.14 (31)	0.00 (0)	0.93	0.07						
g.–377G>T	GG	GT	TT	G	T	489.92	<0.01	0.29	1.53	0.65	0.35
	0.65 (145)	0.26 (58)	0.09 (21)	0.78	0.22						
g.–249G>A	GG	GA	AA	G	A	186.12	<0.01	0.13	1.16	0.86	0.14
	0.85 (191)	0.15 (33)	0.00 (0)	0.93	0.07						

^1^ The number in brackets is the sample size. *PIC*: Polymorphism information content, *Ne*: number of effective alleles, *Ho*: observed heterozygosity, *He*: expected heterozygosity.

**Table 2:** The correlation between four SNPs in the promoter region of the *MyoG* gene and the growth traits of Guizhou White goats.

SNP	Genotype	Body Weight (kg)	Body Length (cm)	Body Height (cm)	Chest Circumference (cm)	Cannon Circumference (cm)
g.–709C>T	CC	26.84 ± 1.08 ^b^	52.17 ± 1.11	58.58 ± 1.23	71.78 ± 1.31 ^b^	7.30 ± 0.15
	CT	29.34 ± 1.06 ^a^	52.52 ± 1.06	59.19 ± 1.22	73.58 ± 1.37 ^a^	7.57 ± 0.14
	TT	27.69 ± 1.23 ^b^	52.41 ± 1.39	59.09 ± 1.40	71.26 ± 1.57 ^b^	7.25 ± 0.21
*p*-value		0.015	0.960	0.848	0.023	0.234
g.–461G>T	GG	28.74 ± 0.94 ^A^	52.47 ± 0.88	58.63 ± 1.45	71.49 ± 1.17	7.46 ± 0.17
	GT	25.14 ± 1.34 ^B^	51.64 ± 1.59	59.19 ± 1.22	69.43 ± 1.73	7.56 ± 0.21
*p*-value		0.001	0.588	0.465	0.162	0.566
g.–377G>T	GG	28.26 ± 0.99	52.28 ± 0.99	54.88 ± 1.23	71.58 ± 1.27	7.44 ± 0.19
	GT	28.14 ± 1.13	53.05 ± 1.09	55.38 ± 1.05	70.27 ± 1.67	7.51 ± 0.15
	TT	27.99 ± 2.41	52.39 ± 1.53	55.98 ± 1.42	69.43 ± 1.74	7.48 ± 0.13
*p*-value		0.893	0.113	0.285	0.162	0.285
g.–249G>A	GG	26.94 ± 2.24	57.39 ± 1.97 ^A^	55.81 ± 1.05	70.89 ± 1.98	7.40 ± 0.12
	AA	24.78 ± 0.93	52.09 ± 1.07 ^B^	59.42 ± 2.09	71.50 ± 1.29	7.52 ± 0.31
*p*-value		0.376	0.008	0.914	0.739	0.566

Note: In the same SNP and column, superscript in different lowercase letters indicates significant difference (*p* < 0.05), and superscript in different uppercase letters indicates extremely significant difference (*p* < 0.01). There is no significant difference in numerical values without letters or with the same letter superscript (*p* > 0.05).

**Table 3:** Alterations of transcription factors before and after SNPs mutation of the *MyoG* gene in Guizhou White goats.

Mutation Location	Nucleotide	Transcription Factors	Transcription Factor Binding Site Nucleotide Sequence	Transcription Factor Location
–709	C	C/EBPalp SRF	tcttacCaaa tcttacCaaaaaagagaa	–715–(–706) –715–(–698)
	T	TBP	Taaaaaagaga	–709–(–699)
–461	G	PU.1	gagGaag	–464–(–458)
	T	AP-1	tatgagTaag	–467–(–458)
–377	G	SP1	cccccaccccacccG	–391–(–377)
	T	OCT-1	Ttcttctttg	–377–(–368)
–249	G	ELA1	Gtcag	–249–(–245)
	A	ALX1	ctaAtcagatta	–252–(–241)

Note: The capital letters in the table indicate mutated bases.

## Data Availability

The original contributions presented in the study are included in the article/Supplementary Materials. Further inquiries can be directed to the corresponding author.

## Supplementary Materials

The following supporting information can be downloaded at: https://www.mdpi.com/article/10.3390/genes17010014/s1, Figure S1: Sanger sequencing chromatograms of different genotype for 4 mutation sites (a–d) in Guizhou White goat *MyoG* gene promoter region. Red rectangles indicate the positions of the mutation sites.
