# Supplementary material for: Single Nucleotide Polymorphisms in the Promoter Region of MyoG Gene Affecting Growth Traits and Transcription Factor Binding Sites in Guizhou White Goat (Capra hircus)
Source: Genes (Basel). 2025 Dec 25;17(1):14. doi: 10.3390/genes17010014 (PMC12841533; doi:10.3390/genes17010014)
Supplement: Supplementary file 1 [file genes-17-00014-s001.zip › genes-4026062-supplementary.pdf]

Supplementary material for:

Single nucleotide polymorphisms in the promoter region of myog gene affecting growth traits and transcription factor binding sites in Guizhou White goat (*Capra hircus*) by Song et al.

a. mutation site g.–709C>T

CC genotype:

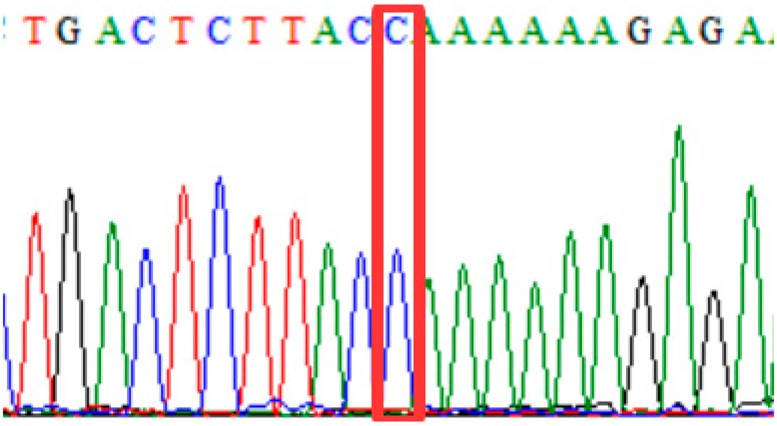

CT genotype:

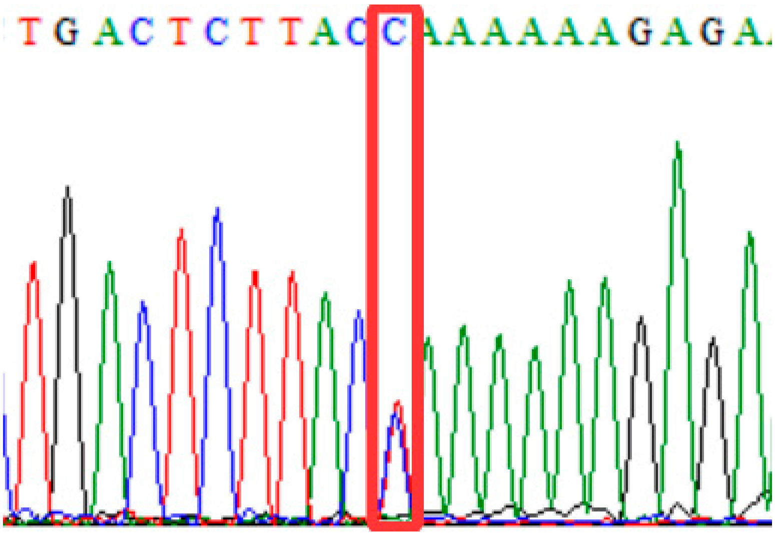

TT genotype:

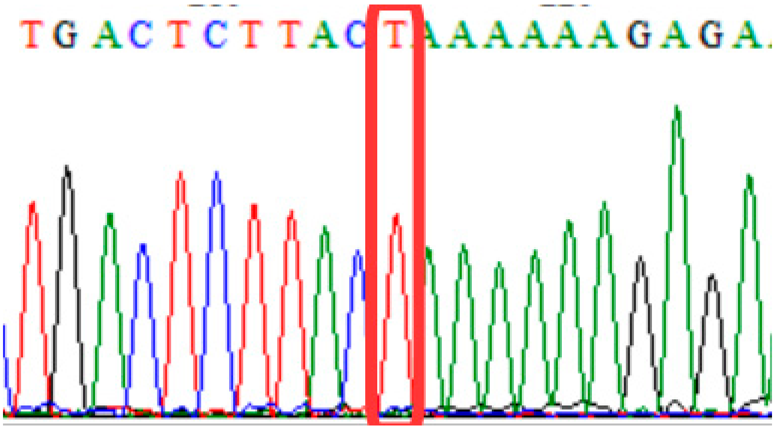

b. mutation site g.–461G>T

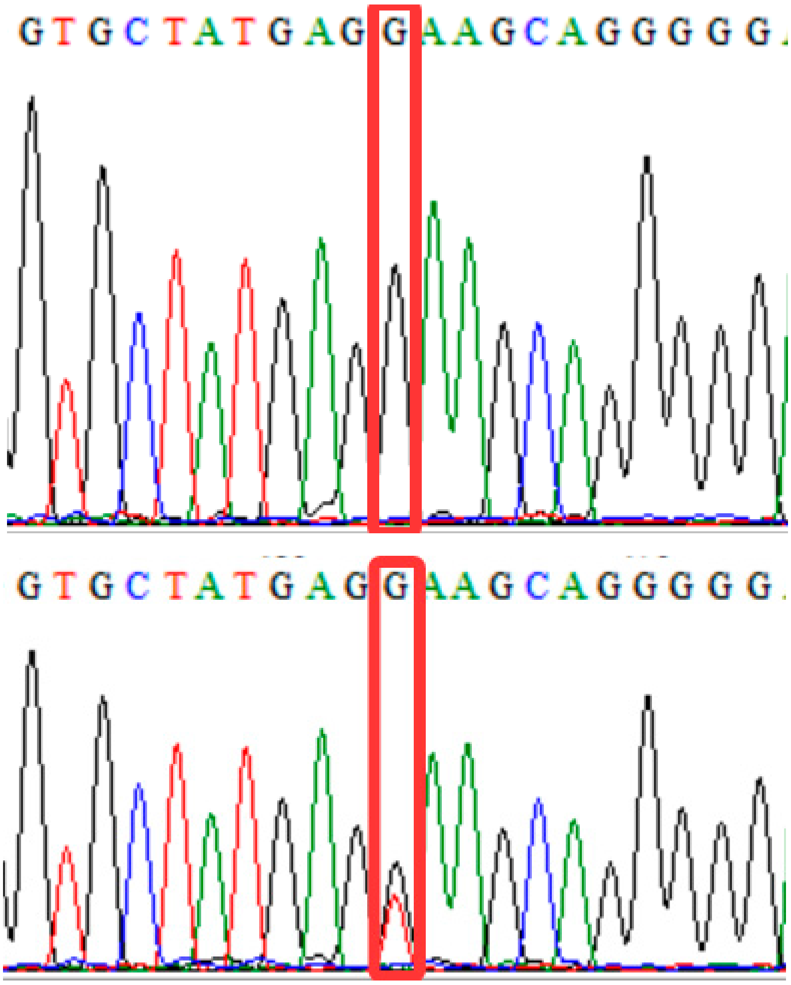

GG genotype:

GT genotype:

c. mutation site g.–377G>T

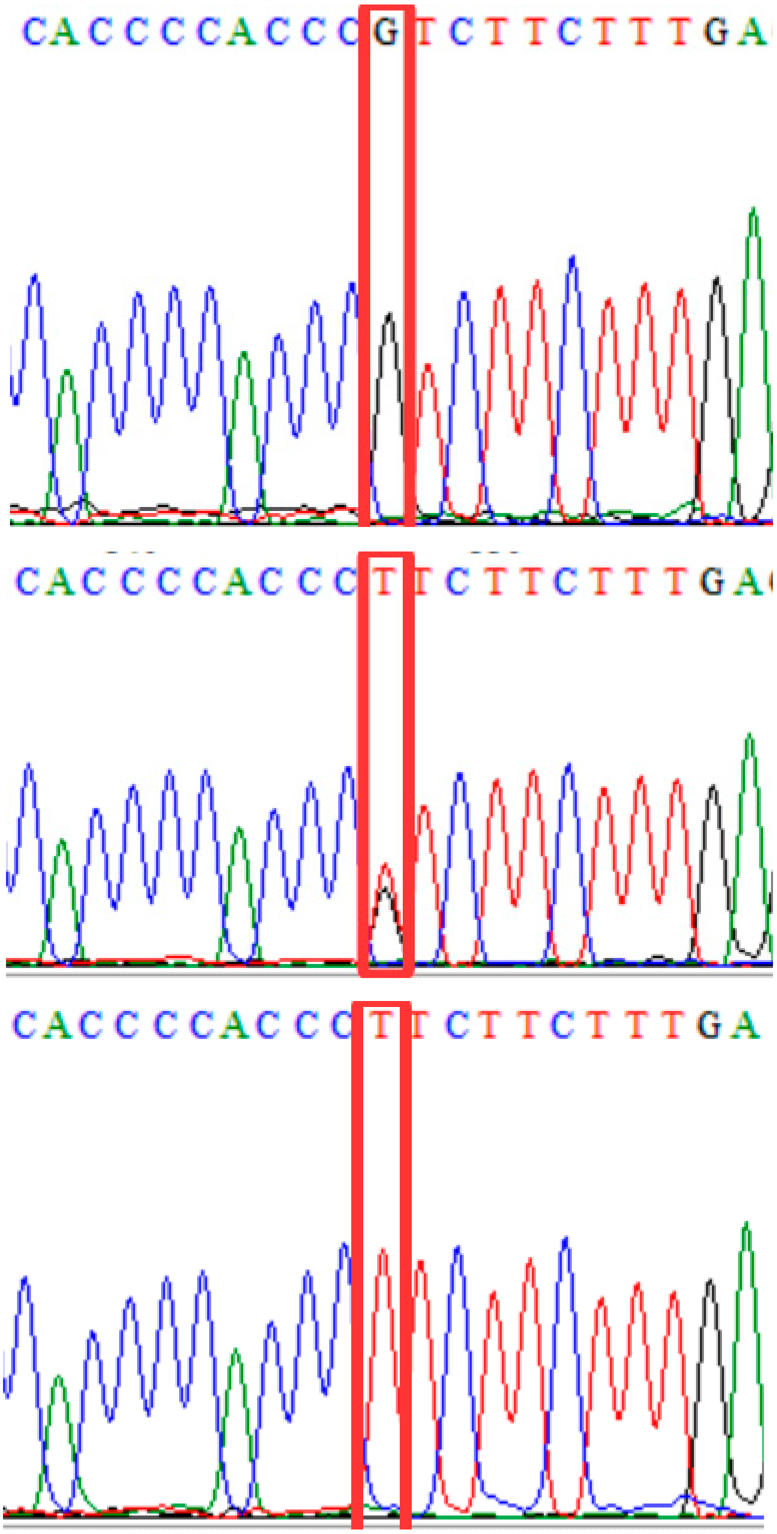

GG genotype:

GT genotype:

TT genotype:

d. mutation site g.–249G>A

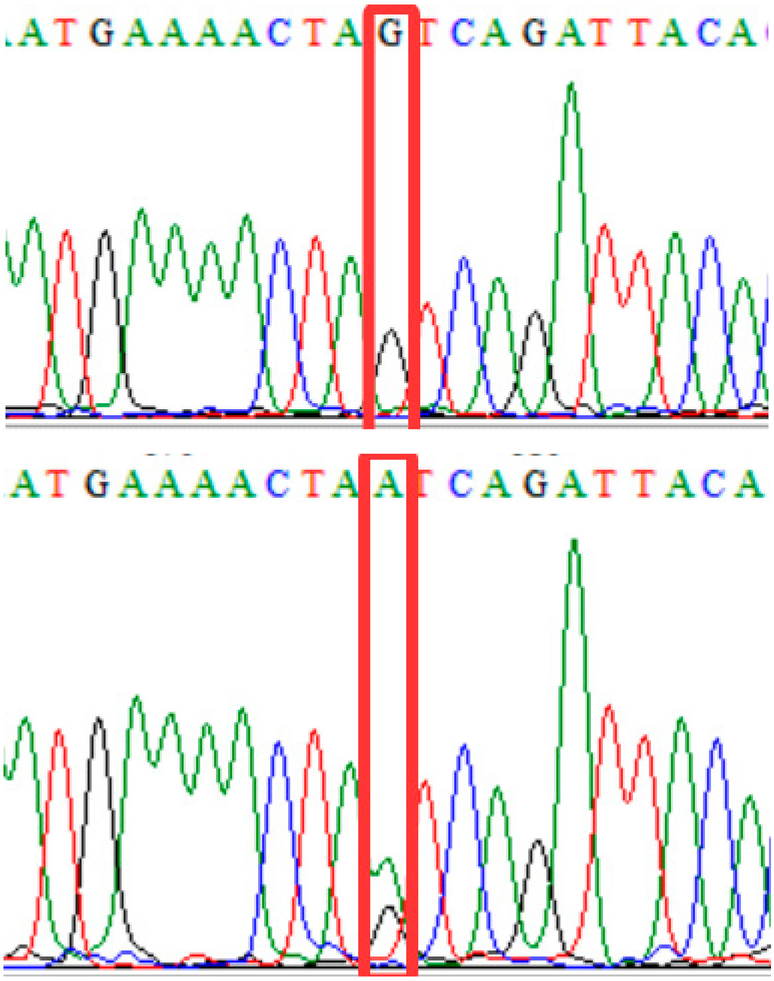

GG genotype:

GA genotype:

Figure S1. Sanger sequencing chromatograms of different genotype for 4 mutation sites(a-d) in Guizhou White goat MyoG gene promoter region. Red rectangles indicate the positions of the mutation sites.
